# Pap testing and high‐risk HPV testing for women aged 65 years and older with surgical pathology follow‐up

**DOI:** 10.1002/cncy.70111

**Published:** 2026-05-04

**Authors:** Timothy G. Ramseyer, Bethany Batson, Daisy Wu, Jonee L. Matsko, Amy K. Colaizzi, Samer N. Khader, Chengquan Zhao

**Affiliations:** ^1^ Department of Pathology University of Pittsburgh Medical Center Pittsburgh Pennsylvania USA

**Keywords:** cervical cancer, cytopathology, human papillomavirus, older women, Papanicolaou test

## Abstract

**Background:**

Current professional guidelines recommend discontinuing Papanicolaou (Pap) test screening in women aged 65 years and older who have had adequate prior negative testing. However, limited data exist on Pap test performance and histologic outcomes in this population.

**Methods:**

Searches were performed for all Pap tests from women aged 65 years and older accessioned at a women’s hospital between January 2023 and December 2024. Pap tests were performed using the liquid‐based cytology ThinPrep test, and high‐risk HPV testing was performed using Aptima high‐risk human papillomavirus (HPV) assays. Surgical pathology follow‐up within 6 months was recorded. A Pearson χ^2^ test was performed to compare HPV positivity among patients who had abnormal Pap tests.

**Results:**

In total, 1536 women aged 65 years and older underwent Pap testing during the study period. The overall HPV‐positivity rate was 24.2%. Abnormal Pap results (atypical squamous cells of undetermined significance or worse) comprised 939 of 1536 cases (61.1%). Histologic follow‐up was available for 402 cases. Lesions categorized as cervical intraepithelial neoplasia grade 2 (CIN2) or more severe disease were identified in 94 cases (23.3%), including 11 squamous cell carcinomas, three endocervical carcinomas, 40 CIN2/3 lesions, and 40 endometrial carcinomas. Notably, three of 11 squamous cell carcinomas (27.3%) and 12 of 40 CIN2/3 lesions (30%) were HPV‐negative.

**Conclusions:**

The abnormal Pap rate in women aged 65 years and older was high (61.1%), whereas HPV positivity remained low. CIN2 or more severe disease and endometrial lesions after negative HPV testing occurred at a substantial rate (33.8%). The rate for detecting atypical glandular cells was also elevated (3.0%), correlating with a significant number of endometrial carcinoma diagnoses. These findings underscore the need for additional research and suggest that continued screening with Pap and HPV cotesting may benefit older women.

## Abstract 2

In a 2‐year review of results from Papanicolaou test cytology and subsequent histology, a substantial proportion of women aged 65 years and older undergoing Papanicolaou testing had abnormal cytology results and significant findings on histologic follow‐up, including high rates of human papillomavirus‐negative grade 2 cervical intraepithelial neoplasia or more severe lesions and endometrial carcinomas, highlighting the potential limitations of discontinuing cervical cancer screening solely based on age.

## INTRODUCTION

Current professional organizations’ guidelines recommend that Papanicolaou (Pap) test screening may be discontinued in women older than 65 years who have had adequate prior negative testing.^1^ According to the 2018 US Preventative Services Task Force cervical cancer screening guidelines, Pap test screening may be discontinued for women older than 65 years if they have had adequate negative prior screening test results, defined as three consecutive negative cytology results, two consecutive negative cotesting results, or two consecutive negative high‐risk human papillomavirus (hrHPV) test results within 10 years.^2^ These guidelines have been endorsed by the American College of Obstetricians and Gynecologists, the American Society of Colposcopy and Cervical Pathology, and the Society of Gynecologic Oncology.^3^ Similarly, the 2020 American Cancer Society cervical cancer screening guidelines recommend discontinuing all cervical cancer screening for women older than 65 years who have no history of cervical intraepithelial neoplasia (CIN) grade 2 or more severe (CIN2+) disease in the prior 10 years and have had documented, adequate, negative prior screening in the prior 10 years.^4^ These guidelines are widely endorsed although an estimated 20% of cervical cancer cases occur in women older than age 65 years in the United States.^5^ In addition, there are relatively few studies that have reviewed the biopsy results after abnormal Pap tests in this patient population to determine the rates of CIN1, CIN2+, and other epithelial abnormalities.

## MATERIALS AND METHODS

After Institutional Review Board approval at the University of Pittsburgh Medical Center, computer‐based searches were performed in the laboratory information system database, Copath (Cerner Corporation), for the results of all Pap tests for women aged 65 years and older accessioned at Magee‐Womens Hospital in a 24‐month period between January 2023 and December 2024. The Magee‐Womens Hospital cytopathology laboratory is within the University of Pittsburgh Medical Center system and is a large, subspecialized, academic hospital laboratory that serves a metropolitan area with a diverse age distribution.^6^ The University of Pittsburgh Medical Center is a large, private health system with a diverse group of clinical providers collecting Pap tests, including gynecologists, family physicians, internists, advanced practice providers, and trainees.

Pap tests were performed using the liquid‐based cytology ThinPrep Pap test (TPPT; Hologic, Inc.) method. TPPTs were prepared according to the manufacturer’s specifications from PreservCyt (Hologic, Inc.) samples using an automated processor (ThinPrep 5000; Hologic, Inc.). TPPT slides were screened with location‐guided, computer‐assisted screening using the ThinPrep Imaging System (Hologic, Inc.).^7^ The ThinPrep Imaging System analyzed batches of up to 250 TPPT slides using specialized imaging software, and the locations of 22 microscopic fields containing cells of interest were recorded. TPPT slides were then placed on cytotechnologist’s ThinPrep Imaging System Review Scopes (Hologic, Inc.) for the cytotechnologists to review the 22 fields. If the cytotechnologist identified no abnormalities in the 22 fields, they could sign out the case results as negative for intraepithelial lesion or malignancy (NILM). If the cytotechnologist identified any abnormality or reactive or reparative changes, the entire TPPT slide was manually rescreened. All cases in which the cytotechnologist identified abnormalities or reactive or reparative changes were evaluated by a cytopathologist.

Results were reported based on the 2014 Bethesda System for reporting cervical cytology.^8^ Interpretation categories included unsatisfactory, NILM, atypical squamous cells (ASC) of undetermined significance (ASCUS), low‐grade squamous intraepithelial lesion (LSIL), atypical squamous cells, cannot exclude high‐grade squamous intraepithelial lesion (ASC‐H), high‐grade squamous intraepithelial lesion (HSIL), squamous cell carcinoma (SCC), atypical glandular cells (AGCs), and adenocarcinoma (AGC+). To consolidate duplicate results, Pap tests with multiple categories of abnormalities (i.e., LSIL and ASC‐H) were assigned to the greater of the two categories (ASC‐H). In cases with squamous and glandular abnormalities in the Pap test results, the cases were assigned to their squamous abnormality category.

hrHPV testing was performed on the Aptima hrHPV testing platform (Gen‐Probe; Hologic, Inc.) according to manufacturer’s specifications. The Aptima HPV assay is a commercially available, US Federal Drug Administration–approved, in vitro nucleic acid amplification test designed to detect HPV E6/E7 messenger RNA from 14 hrHPV types (types 16, 18, 31, 33, 35, 39, 45, 51, 52, 56, 58, 59, 66, and 68).^9^ hrHPV testing was performed based on several ordering options for clinicians: reflex testing after an indeterminate, abnormal, atypical squamous cell (ASC) Pap test result; cotesting with Pap tests in women aged 30 year and older; or cotesting regardless of age or Pap test results. If hrHPV messenger RNA was detected in a patient with a negative Pap test result, the Pap test slides were routinely manually rescreened by the screening cytotechnologist, further manually rescreened by a quality‐assurance cytotechnologist, then reviewed by a cytopathologist.

The results for the surgical pathology specimens in the subsequent 6 months after all abnormal Pap tests were recorded. Surgical pathology follow‐up included cervical biopsy, endocervical curettage, cervical conization by loop electrosurgical excision procedure or cold knife, and hysterectomy specimens. The histologic findings and time elapsed between abnormal Pap test results and follow‐up surgical pathology findings were recorded. For women who had two or more surgical pathology results during the follow‐up period, only the most abnormal histologic diagnosis was recorded.

### Statistical analysis

The Pearson χ^2^ test (IBM SPSS statistical software, version 26.0; IBM Corporation) was used to compare the detection rates of cervical CIN2+ between HPV‐positive and HPV‐negative patients aged 65 years and older who had abnormal Pap tests. In addition, the sensitivity, specificity, positive predictive value, and negative predictive value for detecting cervical CIN2+ lesions and endometrial carcinomas (EM‐ca) in this cohort were calculated with 95% confidence intervals (CIs).

## RESULTS

**FIGURE 1:**
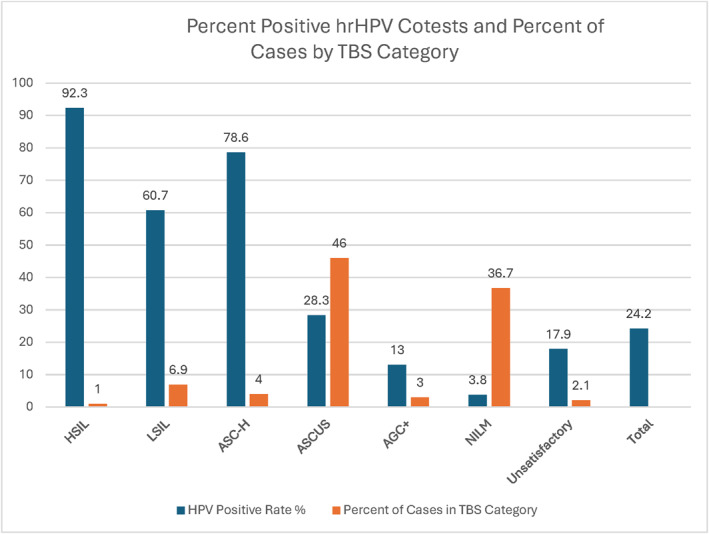
The percentages of Pap tests are illustrated for women aged 65 years and older in each Bethesda category (orange) along with the percentages that were positive for hrHPV on the corresponding cotest (blue). HPV positivity rates ranged from 3.8% for NILM Pap tests to 92.3% for HSIL Pap tests, with an overall HPV positivity rate of 24.2%. In addition, the percentages of Pap tests reported in the atypical categories were higher than typically reported in younger cohorts. AGC+ indicates atypical glandular cells and adenocarcinoma; ASC‐H, atypical squamous cells, cannot exclude high‐grade squamous intraepithelial lesions; ASC‐US, atypical squamous cells of undetermined significance; HPV, human papillomavirus; hrHPV, high‐risk human papillomavirus; HSIL, high‐grade squamous intraepithelial lesion; LSIL, low‐grade squamous intraepithelial lesion; NILM, negative for intraepithelial lesion or malignancy.

During the study period, 1536 women aged 65 year and older underwent Pap testing in our institution, representing 1.9% of all 80,657 Pap tests (Table 1). Abnormal Pap tests (ASCUS or worse) accounted for 61.1% of the total Pap tests (939 of 1536). ASCUS cases accounted for 46.0%, NILM accounted for 36.7%, LSIL accounted for 6.9%, AGC+ accounted for 3.0%, HSIL accounted for 1.0%, and unsatisfactory accounted for 2.1% of Pap tests in this cohort (Figure 1). Concurrent HPV testing was performed in 1451 of 1536 (94.4%) Pap tests. The overall HPV‐positive rate was 24.2% and was highest in the HSIL group (92.3%), and it was lowest in the NILM group (3.8%; Figure 1). The HPV positivity rates were 60.7% in the LSIL group, 78.6% in the ASC‐H group, 28.3% in the ASCUS group, 13.0% in the AGC+ group, and 17.9% in the unsatisfactory group.

**TABLE 1:** Summary of Papanicolaou and human papillomavirus tests for patients aged 65 years and older.

TBS category	No. of cases (%)	Age: Mean, years	No. of HPV tests	No. HPV positive	HPV‐positive rate, %
HSIL	16 (1.0)	72.9	13	12	92.3
LSIL	107 (6.9)	68.5	102	62	60.7
ASC‐H	61 (4.0)	69.9	61	48	78.6
ASCUS	708 (46.0)	69.3	704	199	28.3
AGC+	47 (3.0)	80.0	46	6	13.0
NILM	564 (36.7)	69.4	497	19	3.8
Unsatisfactory	33 (2.1)	70.3	28	5	17.9
Total	1536	69.5	1451	351	24.2

Abbreviations: AGC+, atypical glandular cells and adenocarcinoma; ASC‐H, atypical squamous cells, cannot rule out high‐grade squamous intraepithelial lesion; ASCUS, atypical squamous cells of undetermined significance; HPV, human papillomavirus; HSIL, high‐grade squamous intraepithelial lesion; LSIL, low‐grade squamous intraepithelial lesion; NILM, negative for intraepithelial lesion or malignancy; TBS, The Bethesda System.

Histologic follow‐up was available for 402 of 939 cases reported as ASCUS or greater (42.8%), including 233 with positive HPV tests, 160 with negative HPV tests, and nine without HPV testing (Table 2). CIN2+ high‐grade lesions were identified in 94 of 402 cases (23.3%), including 11 with SCC, three with endocervical carcinoma, 40 with CIN2/3, and 40 with EM‐ca. Among the SCC cases, eight of 11 were HPV‐positive; and, among the EM‐ca cases, all 40 were negative for HPV. Notably, three of 11 SCC cases (27.3%) and 12 of 40 CIN2/3 cases (30%) were HPV‐negative. Among the HPV‐negative cases, CIN2+ high‐grade lesions were identified in 54 of 160 cases (33.8%).

**TABLE 2:** Histologic follow‐up of abnormal Papanicolaou tests for patients aged 65 years and older.

TBS category	HPV test	No. of cases	Biopsy follow‐up: Mean, months	No. (%)					
				EM‐ca	SCC	CIN2/3	EC‐ca	CIN1	Benign
HSIL	HPV+	12	2.0	0 (0.0)	3 (25.0)	5 (42.0)	0 (0.0)	3 (25.0)	1 (8.0)
	HPV−	1	0.0	0 (0.0)	0 (0.0)	0 (0.0)	0 (0.0)	1 (100.0)	0 (0.0)
	NA	3	1.7	0 (0.0)	0 (0.0)	3 (100.0)	0 (0.0)	0 (0.0)	0 (0.0)
LSIL	HPV+	44	4.0	0 (0.0)	0 (0.0)	4 (9.0)	0 (0.0)	26 (59.0)	14 (32.0)
	HPV−	17	3.0	0 (0.0)	0 (0.0)	2 (12.0)	0 (0.0)	7 (41.0)	8 (47.0)
	NA	2	3.5	0 (0.0)	0 (0.0)	1 (50.0)	0 (0.0)	1 (50.0)	0 (0.0)
ASC‐H	HPV+	36	1.9	0 (0.0)	2 (6.0)	11 (31.0)	0 (0.0)	19 (53.0)	4 (11.0)
	HPV−	10	1.3	0 (0.0)	0 (0.0)	2 (20.0)	0 (0.0)	3 (30.0)	5 (50.0)
	NA	0	NA						
ASCUS	HPV+	135	3.2	0 (0.0)	3 (2.0)	3 (2.0)	0 (0.0)	85 (63.0)	44 (33.0)
	HPV−	93	3.3	11 (12.0)	3 (3.0)	8 (9.0)	0 (0.0)	20 (22.0)	51 (55.0)
	NA	3	4.7	0 (0.0)	0 (0.0)	1 (33.0)	0 (0.0)	0 (0.0)	2 (66.0)
AGC+	HPV+	6	4.6	0 (0.0)	0 (0.0)	0 (0.0)	3 (50.0)	0 (0.0)	3 (50.0)
	HPV−	39	1.1	28 (72.0)	0 (0.0)	0 (0.0)	0 (0.0)	1 (3.0)	10 (26.0)
	NA	1	1.6	1 (100.0)	0 (0.0)	0 (0.0)	0 (0.0)	0 (0.0)	0 (0.0)
Summary	HPV+	233	3.1	0 (0.0)	8 (3.0)	23 (10.0)	3 (1.0)	133 (57.0)	66 (28.0)
	HPV−	160	2.5	39 (24.0)	3 (2.0)	12 (8.0)	0 (0.0)	32 (20.0)	74 (46.0)
	NA	9	2.5	1 (11.0)	0 (0.0)	5 (55.0)	0 (0.0)	1 (11.0)	2 (22.0)

Abbreviations: −, negative; +, positive; AGC+, atypical glandular cells and adenocarcinoma; ASCUS, atypical squamous cells of undetermined significance; CIN2/3, cervical intraepithelial neoplasia 2 or 3; EC‐ca, endocervical adenocarcinoma; EM‐ca, endometrial carcinoma; HPV, human papillomavirus; HSIL, high‐grade squamous intraepithelial lesion; LSIL, low‐grade squamous intraepithelial lesion; NA, not available; SCC, squamous cell carcinoma; TBS, The Bethesda System.

HSIL cases accounted for 1% of the total Pap tests (16 of 1536 tests). Histologic follow‐up was available for 16 cases (100%), including 12 with positive HPV tests, one with a negative HPV test, and three with no HPV testing. Among the 12 HPV‐positive cases in this group, three had SCC, five had CIN2/3, three had CIN1, and one had benign histologic follow‐up. The HPV‐negative case had CIN1 on histologic follow‐up, and all three of the Pap tests with no HPV testing had CIN2/3 on histologic follow‐up.

LSIL cases accounted for 6.9% of the total Pap tests (107 of 1536 tests). Histologic follow‐up was available for 63 cases (58.9%), including 44 with positive HPV tests, 17 with negative HPV tests, and two with no HPV testing. Among the 44 HPV‐positive cases in this group, 26 had CIN1, 14 had benign findings, and four had CIN2/3 on histologic follow‐up. Among the 17 cases with negative HPV tests, eight had benign findings, seven had CIN1, and two had CIN2/3 on histologic follow‐up. One of the two cases with no HPV testing had CIN2/3 on histologic follow‐up, and the other had CIN1.

ASC‐H cases accounted for 4.0% of the total Pap tests (61 of 1536 tests). Histologic follow‐up was available for 46 cases (75.4%), including 36 with positive HPV tests and 10 cases with negative HPV tests. Among the 36 cases with positive HPV tests, 19 had CIN1, 11 had CIN2/3, four had benign findings, and two had SCC on histologic follow‐up. Among the 10 cases with negative HPV tests, five had benign findings, three had CIN1, and two had CIN2/3 on histologic follow‐up.

ASCUS cases accounted for 46% of the total Pap tests (708 of 1536 tests). Histologic follow‐up was available for 231 cases (32.6%), including 135 with positive HPV tests, 93 with negative HPV tests, and three with no HPV testing. Among the 135 cases with positive HPV tests, 85 had CIN1, 44 had benign findings, three had CIN2/3, and three had SCC on histologic follow‐up. Among the 93 cases with negative HPV tests, 51 had benign findings, 20 had CIN1, 11 had endometrial adenocarcinoma, eight had CIN2/3, and three had SCC on histologic follow‐up. Among the three cases with negative HPV tests, two had benign findings on histologic follow‐up, and one had CIN2/3 on histologic follow‐up.

AGC+ cases accounted for 3.0% of the total Pap tests (47 of 1536 tests). Histologic follow‐up was available for 46 cases (97.8%), including six with positive HPV tests, 39 with negative HPV tests, and one with no HPV testing. Among the six cases with HPV tests, three had endocervical adenocarcinoma, and the remaining three had benign findings on histologic follow‐up. Among the 39 HPV‐negative, AGC+ Pap tests, 28 had endometrial adenocarcinoma, 10 had benign findings, and one had CIN1 on histologic follow‐up. The case with no HPV testing had endometrial adenocarcinoma on histologic follow‐up.

The performance of HPV testing to detect cervical high‐grade lesions, endocervical adenocarcinoma, and EM‐ca was assessed in the patients who had abnormal Pap results. Among 233 HPV‐positive cases, 34 (14.6%) had cervical CIN2+ lesions (HSIL, SCC, or endocervical adenocarcinoma) on histologic follow‐up. Among 160 HPV‐negative cases, 15 (9.4%) had cervical CIN2+ lesions on histologic follow‐up. The difference in detecting cervical CIN2+ lesions indicated no statistical significance between the HPV‐positive and HPV‐negative groups (*p* = .570). Next, we analyzed the value of HPV testing to detect cervical CIN2+ lesions and EM‐ca. Among 233 HPV‐positive cases, 34 (14.6%) had cervical CIN2+ lesions, and none had EM‐ca. Among 160 HPV‐negative cases, 54 (33.8%) had cervical CIN2+ lesions and EM‐ca on histologic follow‐up. The difference in detecting cervical CIN2+ lesions and EM‐ca indicated statistical significance between the HPV‐positive and HPV‐negative groups (*p* < .001). The performance of the HPV test in detecting cervical high‐grade lesions and EM‐ca is detailed in Table 3.

**TABLE 3:** Diagnostic value of positive human papillomavirus status for detecting cervical high‐grade squamous lesions or more severe disease and endometrial carcinomas in patients aged 65 years and older with abnormal Papanicolaou tests.

	Sensitivity (95%CI), %	Specificity (95% CI), %	PPV (95%CI), %	NPV (95% CI), %
CIN2+	69.4 (54.4–81.3)	34.8 (29.5‐40.4)	14.6 (10.4–19.9)	87.6 (80.1–92.7)
CIN2+ and EM‐ca,	38.6 (28.6–49.7)	34.8 (29.5–40.4)	14.6 (10.4–19.9)	66.3 (58.3‐73.4)

Abbreviations: CI, confidence interval; CIN2+, cervical intraepithelial neoplasia grade 2 or more severe disease (cervical high‐grade squamous lesions, endocervical adenocarcinoma, and squamous cell carcinoma); EM‐ca, endometrial carcinoma; NPV, negative predictive value; PPV, positive predictive value.

## DISCUSSION

In the current study, the overall HPV‐positive rate was 24.2%, and the rate of squamous lesions on cases with histologic follow‐up was 53.9%. In this cohort, HPV positivity was associated with squamous lesions and endocervical adenocarcinomas on histologic follow‐up. HSIL Pap tests were most associated with high‐grade squamous lesions or SCCs on histologic follow‐up (67%). The rate of high‐grade squamous lesions or SCCs on histologic follow‐up was 7.8% after ASCUS Pap tests, 11.1% after LSIL Pap tests, and 32.6% after ASC‐H Pap tests. In contrast, HPV negativity was associated with glandular lesions on histologic follow‐up, with a high number of EM‐ca identified on histologic follow‐up in this cohort (40), including 29 after AGC+ Pap tests and 11 after HPV‐negative ASCUS Pap tests.

The rates of squamous lesions on histologic follow‐up after abnormal squamous findings on Pap tests in this study were higher than the rates reported in previous studies.^10^, ^11^, ^12^ For instance, the rate of CIN1 after an ASCUS Pap test was 67% with positive hrHPV cotesting in our cohort; and, in a prior study from our institution that included younger women, the rate of CIN1 after ASCUS Pap tests with positive hrHPV cotesting ranged from 41.1% in the group aged 40–49 years to 49.2% in the group aged 60 years and older.^10^ In the current cohort, the rates of CIN2+ lesions after Pap tests with positive HPV testing were similar to or higher than the rates reported in younger cohorts.^10^, ^12^ For instance, in a previous study at our institution, which included younger women, that examined LSIL Pap tests performed between 2013 and 2017, the rate of CIN2/3 on histologic follow‐up was 8.9%; and, in our current cohort, the rate was 9.0%.^12^

Although the current guidelines in the United States suggest that Pap test screening may be discontinued for women older than 65 years who have had appropriate previous screening, these recommendations may need to be reconsidered because the evidence regarding the incidence of HPV infections and cervicovaginal squamous lesions in older women changes. In a 2023 study of the German Center of Cancer Registry data from 2001 to 2015, Neumeyer et al. observed that 27.6% of cervical cancers occurred in women older than 65 years and that 5‐year survival was worse for women in this age group.^13^ Similarly, in a 2022 study comparing the Pap test records from 2008 to 2018 for Korean women aged 65 years and older versus women younger than 65, Cho et al. observed that the incidence of HSIL or worse was significantly higher in the group aged 65 years and older, with a statistically significantly increased number of HPV‐negative HSIL cases.^14^ In current our study, the percentage of women with HSIL Pap tests was 1.0%, and the overall rate of squamous lesions identified on histologic follow‐up was 53.9%, which are similar to or higher than the rates that have been reported in younger cohorts.^15^, ^16^, ^17^, ^18^ Although these cohorts of older women may be enriched for women who are at higher risk of HPV‐related squamous lesions, the overall findings suggest that individualized risk assessment is important to ensure adequate screening for older women in the United States.

Notably, our cohort included three histologically diagnosed SCCs after HPV‐negative ASCUS Pap tests. Previous studies have reported hrHPV‐negative HSIL rates of between 4.2% and 8.3%, with HPV‐negative squamous lesions occurring more frequently in older women.^19^, ^20^, ^21^ Because hrHPV prevalence declines with increasing age and hrHPV positivity is strongly associated with squamous lesions on histology, this age‐related decrease in HPV detection has been cited as a rationale for current guidelines recommending cessation of Pap test screening in women aged 65 years and older.^22^, ^23^, ^24^, ^25^ Although the number of HPV‐negative SCCs was low in this cohort, our results suggest that reliance on hrHPV testing alone may miss a clinically meaningful subset of high‐grade squamous lesions and invasive carcinomas in older women. In this study, 27.3% of SCCs and 30% of CIN2/3 lesions were HPV‐negative, a proportion that is higher than typically reported in younger populations.^10^, ^11^, ^12^ Several mechanisms have been proposed to explain HPV‐negative squamous lesions in older women, including a low viral load below assay detection thresholds, loss of detectable viral nucleic acid during tumor progression, infection with non‐hrHPV types, or non–HPV‐driven carcinogenic pathways.^19^, ^20^, ^21^

In addition to squamous lesions, our study highlights the important role of Pap testing in identifying glandular and noncervical malignancies in women aged 65 years and older. The AGC+ rate in this cohort (3.0%) was substantially higher than that reported in most screening populations, and histologic follow‐up revealed a strikingly high prevalence of EM‐ca, particularly among HPV‐negative cases.^26^ Among HPV‐negative, AGC+ Pap tests, 72% were associated with endometrial adenocarcinoma on follow‐up. Notably, EM‐ca was also identified after HPV‐negative ASCUS Pap tests, underscoring the value of cytologic screening in detecting clinically significant, gynecologic, noncervical pathology in this age group. These findings support prior observations that glandular abnormalities in older women warrant thorough evaluation regardless of HPV status.^26^, ^27^

The high abnormal Pap rate observed in this cohort (61.1%), driven largely by the ASCUS and AGC categories, differs markedly from distributions reported in younger screening populations.^10^, ^11^, ^12^ Although some of this increase may reflect age‐related cytomorphologic changes, such as atrophy and inflammation, the substantial proportion of cases with significant histologic abnormalities suggests that these cytologic interpretations cannot be dismissed as benign, age‐related artifacts. It is important to note that nearly one half of ASCUS cases with histologic follow‐up harbored CIN1 or more severe pathology, and 7.8% demonstrated CIN2+ or SCC, reinforcing the clinical relevance of ASCUS interpretations in older women.

In addition to Pap testing, hrHPV genotyping and age‐adjusted risk stratification have been proposed as methods to assist triaging within national cervical cancer screening programs.^28^ The persistence of infection and the risk of developing cervical cancer varies greatly between HPV types, and HPV16 infection is associated with the highest risk of cervical cancer followed by HPV18.^29^ In a recent study evaluating genotype‐specific clearance rates and subsequent cervical disease risks for 14 persistent hrHPV genotypes among women with mild cytology results (NILM or ASCUS) in Finland, Numminen et al. observed that persistent infections with HPV types 16, 58, 33, and 52 were associated with the highest risks of progression to high‐grade squamous lesions and that younger women infected by these HPV types had higher rates of progression to high‐grade squamous lesions.^30^ In a similar study of Pap screening data from Finland, Leino et al. reported that, among 6031 hrHPV‐positive women, the prevalence of high‐grade squamous lesions  was highest in HPV16‐positive women (37.3%), followed by HPV18 (26.0%), and other HR‐HPV types (20.3%), and the detection of high‐grade squamous lesions declined with age.^28^ The results from these studies suggest that younger women and women with HPV16 infections should be prioritized for follow‐up screening and subsequent tissue sampling. In contrast, the high rates of squamous lesions and adenocarcinomas after hrHPV‐negative Pap testing in our cohort highlight the importance of Pap testing for older women. Among women with abnormal Pap tests in our cohort, hrHPV testing had relatively low sensitivity for squamous lesions and endocervical adenocarcinomas, with even lower sensitivity when EM‐ca was included. Although the HPV genotype data were not available for this cohort, these findings suggest that Pap test cytology detects a meaningful number of HPV‐negative malignancies that might be missed if Pap testing is deprioritized in this age group.

Several limitations of this study should be acknowledged. This was a single‐institution, retrospective analysis conducted at a large tertiary care academic center, which may introduce referral and selection biases and limit generalizability. Only 42.8% of abnormal Pap tests had histologic follow‐up within 6 months, potentially underestimating or overestimating true lesion prevalence. In addition, detailed prior screening histories were not uniformly available, precluding stratification based on adequacy of previous negative screening, which is a key determinant in current guideline recommendations. Despite these limitations, the large sample size, the high rate of HPV cotesting, and the comprehensive histologic follow‐up provide valuable insight into the performance of Pap and HPV testing in this understudied population.

Overall, our findings add to a growing body of evidence suggesting that women aged 65 years and older remain at risk for clinically significant cervical and endometrial pathology, including HPV‐negative, high‐grade squamous lesions and invasive carcinomas. As population demographics shift and life expectancy increases, the balance between screening benefits and harms in older women warrants continued reassessment.

## CONCLUSION

In this large institutional cohort of women aged 65 years and older, abnormal Pap tests were common and frequently associated with clinically significant histologic findings. Although overall hrHPV positivity was relatively low, a substantial proportion of high‐grade squamous lesions and SCCs were HPV‐negative. In addition, Pap testing—particularly in cases interpreted as AGC+ or HPV‐negative ASCUS—identified high numbers with EM‐ca highlighting the broader diagnostic value of cytologic screening in older women. These findings suggest that discontinuation of cervical cancer screening based solely on age and prior screening history may fail to identify important pathology in a subset of women. In addition, the low sensitivity of hrHPV testing among women with abnormal Pap tests in this cohort suggests that Pap and HPV cotesting may detect a meaningful number of squamous lesions and adenocarcinomas in this age group that would be missed by hrHPV screening only. Continued Pap testing with HPV cotesting and individualized risk‐based assessment may provide meaningful clinical benefit for selected women older than 65 years. Further multicenter and prospective studies are needed to refine screening strategies and guideline recommendations for this growing population.

## AUTHOR CONTRIBUTIONS

**Timothy G. Ramseyer:** Conceptualization; data curation; investigation; validation; visualization; and writing—original draft. **Bethany Batson:** Investigation; data curation; validation; and writing—review and editing. **Daisy Wu:** Investigation; data curation; validation; and writing—review and editing. **Jonee L. Matsko:** Investigation; validation; and writing—review and editing. **Amy K. Colaizzi:** Investigation; validation; and writing—review and editing. **Samer N. Khader:** Investigation; validation; and writing—review and editing. **Chengquan Zhao:** Conceptualization; data curation; validation; writing—review and editing; supervision.

## CONFLICT OF INTEREST STATEMENT

The authors declare no conflicts of interest.
